# Effects of Coated Sodium Butyrate on Growth Performance and Intestinal Morphology and Microbiota of White King Pigeon at Weaning Transition

**DOI:** 10.3390/vetsci13080744

**Published:** 2026-07-27

**Authors:** Zhen Liu, Ying Bao, Tiantian Gu, Li Chen, Tao Zeng, Lizhi Lu, Zhizhong Lin

**Affiliations:** 1 College of Agriculture, Yanbian University, Yanji 133000, China; 2 Institute of Animal Husbandry and Veterinary Science, Zhejiang Academy of Agricultural Sciences, Hangzhou 310021, China; 3 Center of Animal Husbandry Development, Rural Bureau of Aagrigriculture of Zhejiang Kaihua County, Quzhou 324300, China

**Keywords:** coated sodium butyrate, White King pigeons, weaning transition, intestinal health

## Abstract

The current study aims to assess the effects of coated sodium butyrate (CSB) on the weaning transition of White King pigeons. A total of 336 1-month-old White King pigeons were assigned randomly to four groups, including the Control group fed basal health sandand without CSB and test groups fed the basal health sand with 0.1%, 0.2%, and 0.4% CSB, respectively. The results showed that, compared with the Control group, the average daily feed intake (ADFI) was significantly increased in the 0.1% and 0.2% CSB groups (*p* < 0.05). The 0.1% CSB group exhibited a higher immune organ index (IOI) than the Control group (*p* < 0.05). The total protein (TP) and albumin (ALB) levels in the 0.1% CSB and 0.4% CSB groups significantly increased compared with the Control group (*p* < 0.05). In contrast, the 0.2% CSB group exhibited lower serum ALB, TP, and total cholesterol (TC) levels; TNF-α and IL-6 concentrations were reduced compared with the other groups (*p* < 0.05), and the 0.4% CSB group showed significantly increased serum TNF-α, IL-6, and D-lactic acid levels (*p* < 0.05). The ratio of villus height to crypt depth (VH/CD) of the jejunum (*p* < 0.05) was significantly increased in the 0.2% CSB group. In addition, the CD and VH/CD of the ileum were significantly increased in the 0.1% CSB group (*p* < 0.05). Firmicutes was the predominant phylum across all treatment groups, while Bacteroidota showed a more sensitive response to CSB supplementation. The 0.1% CSB group showed a significant enrichment of Oscillospiraceae and Muribaculaceae, fiber-fermenting, SCFA-producing taxa, Enterobacterales and Klebsiella, suggesting CSB-mediated alterations in luminal oxygen tension that favor the coexistence of facultative and obligate anaerobic taxa. Collectively, these data revealed that CSB supplementation efficiently improves growth performance and intestinal health of weaning pigeons by ameliorating the intestinal environment.

## Simple Summary

Squabs receive crucial nutrition from “pigeon milk” until 1 month of age, but the experience of the weaning transition is rarely reported, and finding ways to decrease its influence is essential for maximizing the economic benefits of feeding. This study assessed whether the weaning transition could influence the growth performance of pigeons and examined the role of coated sodium butyrate (CSB) in mitigating this effect. In this study, CSB supplementation efficiently improves growth performance and intestinal health of weaning pigeons by ameliorating the intestinal environment.

## 1. Introduction

Pigeon meat is widely recognized as a high-quality protein source with a meat protein content of 67.8%, exceeding that of pork, beef, mutton, and chicken while also demonstrating superior digestibility in comparative studies [1]. Globally, pigeons hold significant economic value for meat production, with China, Egypt, and Iran being the leading producers. China alone accounts for approximately 80% of global pigeon meat production, with an annual production of approximately 680 million pigeons [2]. The scale of this industry makes it essential to optimize growth performance and health management in pigeon farming to maintain both productivity and product quality. The White King pigeon (WK pigeon) is one of the most widely bred pigeon varieties, and the one-month-old stage represents a critical transition period from parental feeding to self-feeding with physiological maturation. During this window, the development of the digestive and reproductive systems accelerates, and nutritional requirements increase substantially to support rapid growth. Research has shown that inadequate nutrition during this critical period can lead to persistent negative effects on growth trajectories, gut health, disease resistance, and long-term reproductive performance, including reduced egg production later in adulthood [3]. Since the 1940s, the agricultural industry has relied heavily on antimicrobial growth promoters (AGPs) to enhance livestock growth rates, improve feed conversion efficiency, and prevent disease [4]. However, the prolonged use of AGPs has posed serious public health threats—drug-resistant pathogens can be transmitted across the food chain between animals and humans [5]. In response, many countries have progressively implemented bans on antibiotics in animal feed, underscoring the urgent need to develop safe and effective alternatives that maintain animal health and production performance without exacerbating antimicrobial resistance [6].

CSB offers improved storability and is devoid of the pungent odor characteristic of uncoated sodium butyrate. CSB supplementation has shown potential in promoting animal growth and development, intestinal health, and immune function in livestock, particularly in weaned piglets, broilers, and dairy cattle, in which it enhances gut barrier integrity, modulates gut microbiota composition, and improves growth performance [7,8]. Health sand, composed of grit, shells, minerals, and vitamins, is essential for maintaining pigeon health. It supplies critical minerals that facilitate digestion, enhance nutrient absorption, and satisfy the bird’s nutritional needs. Insufficient supplementation predisposes pigeons to digestive disorders and increased mortality. The comparable particle sizes of CSB and health sand enable homogeneous blending and co-administration.

This research investigates the impact of CSB on growth performance, serum biochemical indices, intestinal development, microbiota, and immune function of WK pigeons. The primary objective is to assess the efficacy of CSB in mitigating the negative effects of the weaning transition and to provide a scientific foundation for its application as a feed additive in the pigeon industry.

## 2. Materials and Methods

### 2.1. Animals and Diets, and Experimental Design

The animals in this study were bred and euthanized following the National Standard of Laboratory Animal Guidelines for Ethical Review (GB/T 35892-2018) from China. All procedures were approved by the Institute of Animal Husbandry and Veterinary Science, Zhejiang Academy of Agricultural Sciences. A total of 336 healthy 1-month-old 353.90 ± 19.41 g body weight WK pigeon birds were divided into 4 groups, 7 replicates per group, each replicate containing 12 birds with similar performance (gender is random) from a commercial pigeon farm (Huzhou Huajia Special Breeding Co., Ltd., Huzhou, China). Birds in the Control, 0.1% CSB, 0.2% CSB, and 0.4% CSB groups were fed with basal health sand without CSB (Control), basal health sand supplemented with 0.1% CSB additive (0.1% CSB), basal health sand supplemented with 0.2% CSB, and basal health sand supplemented with 0.4% CSB, respectively. The CSB used in our experiment is You Puding, which was kindly provided by Hangzhou King Techina Feed Co. Ltd., Hangzhou, China. You Puding is a commercial product with sodium butyrate in the coating technique (purity = 30%). The pigeons were housed in three-layer cages, environmentally controlled, with every day having 10 h of artificial illumination. According to the farming methods, diets used by the pigeon industry were formulated based on pean-corn-sorghum and wheat. The exact composition of the experimental diets is shown in Table 1. The whole experiment period lasted 4 months.

### 2.2. Growth Performance Analysis

At the end of 1 month and 5 months of age, the body weights (BWs) of WK pigeons were measured. Feed intake per cage was recorded daily. The Average Daily Gain (ADG) and Average Daily Feed Intake (ADFI) were quantified through the analysis of data collected from each individual cage.

### 2.3. Serum Biochemical Analysis

After a 12 h fast, four pigeons from each group, closest to the average body weight, were selected for blood collection from the carotid artery at five-months old. Serum was separated by centrifuging at 3000× *g* for 15 min at 4 °C using an Eppendorf 5424R centrifuge (Eppendorf Co., Ltd., Hamburg, Germany), and then stored at −20 °C for later analysis. The levels of alanine aminotransferase (ALT), alkaline phosphatase (ALP), total serum cholesterol (TC), serum albumin (ALB), total protein (TP), triglyceride (TG), and aspartate aminotransferase (AST) were measured in accordance with the manufacturer’s instructions, utilizing the appropriate reagent kits provided by Nanjing Jiancheng Bioengineering Institute (Nanjing, China).

### 2.4. Immune Organ Index

At the conclusion of the experiment, the pigeons were humanely euthanized via cervical dislocation, in strict adherence to the animal welfare regulations governing slaughter in China. The masses of the pigeons’ thymus and spleen were determined. Subsequently, the thymus index, spleen index, and bursal index were calculated using the obtained data. The immune organ index (IOI) (mg/g) = the immune organs’ weight divided by the live weight of the pigeon on an empty stomach before slaughter.

### 2.5. Inflammatory Factors Analysis

The concentrations of interleukin-6 (IL-6), tumor necrosis factor-α (TNF-α), and D-lactic acid (DL-A). Enzyme-linked immunosorbent assays (ELISAs) were conducted utilizing kits procured from Beijing Huaying Biological Technology Development Co. Ltd. (Beijing, China), following the protocols specified by the manufacturer.

### 2.6. Small Intestinal Histomorphology Analysis

Three parts of the small intestine, the duodenum (from the stomach to pancreaticobiliary duct), jejunum (from pancreaticobiliary duct to the ileocecal junction), and ileum (from the ileocecal junction to ileocecal valve), were each approximately 2–3 cm. The samples were separated, washed with phosphate-buffered saline, dehydrated through a graded ethanol series, and embedded in paraffin as described by Vargas [9]. To measure the morphology of intestinal tissue, the target area of each tissue section was imaged at 40× magnification using an Eclipse Ci-L photomicroscope(Nikon Corporation, Tokyo, Japan) with consistent background lighting for each photograph. After imaging, measurements were taken of five complete villi lengths, five single villi cup cell counts, five crypt depths, and five mucosal thicknesses in each section separately. The Image-Pro Plus 6.0 analysis software was used, with microns serving as the standard unit, to obtain these measurements. Finally, the average values were calculated.

### 2.7. Microbial 16S rRNA Analysis of Ileum Digesta

The ileum of each bird was isolated, and chyme samples were stored at −80 °C for later analysis. To explore the microbial composition and changes in ileum microbiota among different groups, 16S rDNA sequencing technology was employed [10]. Microbial DNA from pigeon ileum samples was extracted using the E.Z.N.A.^®^ Soil DNA Kit (Omega Bio-tek, Inc., Norcross, GA, USA). The V4–V5 region of the bacterial 16S rRNA gene was PCR-amplified with an initial denaturation at 95 °C for 2 min, followed by 25 cycles of 95 °C for 30 s, 55 °C for 30 s, and 72 °C for 30 s, and a final extension at 72 °C for 5 min. Primers 515F (5′-barcode-GTGCCAGCMGCCGCGG-3′) and 907R (5′-CCGTCAATTCMTTTRAGTTT-3′) were used, with each sample having a unique eight-base barcode. Polymerase chain reaction (PCR) assays were conducted in triplicate, utilizing 20 μL reaction mixtures. Each mixture comprised 4 μL of 5 × FastPfu Buffer, 2 μL of 2.5 mM deoxynucleotide triphosphates (dNTPs), 0.8 μL of each primer (5 μM), 0.4 μL of FastPfu Polymerase (TransGen Biotech Co., Ltd., Beijing, China), and 10 ng of template DNA. The resulting amplicons were separated on 2% agarose gels and subsequently purified using the AxyPrep DNA Gel Extraction Kit (Axygen Biosciences, Union City, CA, USA) in accordance with the manufacturer’s instructions. One sample in the 0.2% group was excluded due to sampling error/death, resulting in 5, 5, 4, and 5 replicates for the Control, 0.1% CSB, 0.2% CSB, and 0.4% CSB groups, respectively.

Raw paired-end reads were quality-filtered using Fastp and merged with FLASH. High-quality sequences were clustered into operational taxonomic units (OTUs) at 97% sequence similarity using UPARSE, and representative sequences were taxonomically assigned against the SILVA 138 database using the RDP Classifier (confidence threshold = 80%). Downstream analyses were performed using QIIME2 (v2019.1). Alpha diversity was evaluated using the Chao1, ACE, Shannon, and Simpson indices, while beta diversity was assessed by principal coordinate analysis (PCoA) based on Bray–Curtis distances, with group differences tested by one-way ANOVA. Differential taxa were identified using LEfSe (LDA score > 3.0), and microbial functional profiles were predicted using PICRUSt2 based on KEGG pathway annotation.

### 2.8. Statistical Analysis

Office and SPSS 20.0 software were used for one-way ANOVA and data processing. Regression and statistical analyses were performed, with results expressed as means ± SEM. Significant effects in ANOVA were further analyzed using Duncan’s multiple range test, with *p*-values < 0.05 deemed significant. During the preparation of this manuscript, the author (s) used ChatGPT (OpenAI, GPT-4) solely for language polishing purposes, including grammar correction, spelling, punctuation, and formatting refinement. No generative AI tools were used for study design, data collection, data analysis, interpretation of results, or figure/table generation. The author (s) take full responsibility for the intellectual content and integrity of this work.

## 3. Results

### 3.1. Growth Performance

As presented in Table 2, the initial BW of pigeons did not differ significantly among the four groups (*p* > 0.05). However, after a 4-month breeding experiment, from 1 to 5 months of age, the ADFI was significantly higher in the 0.1% CSB and 0.2% CSB groups than in the Control and the 0.4% CSB groups (*p* < 0.05). Moreover, the 0.1% CSB group exhibited the greatest ADG, as evidenced by a statistically significant difference compared to the Control group (*p* < 0.05). Pigeons in the 0.1% CSB group exhibited a statistically significant increase in final BW (*p* < 0.05) and ADG (*p* < 0.05) as compared with those in other groups.

### 3.2. Serum Biochemical

**Figure 1:**
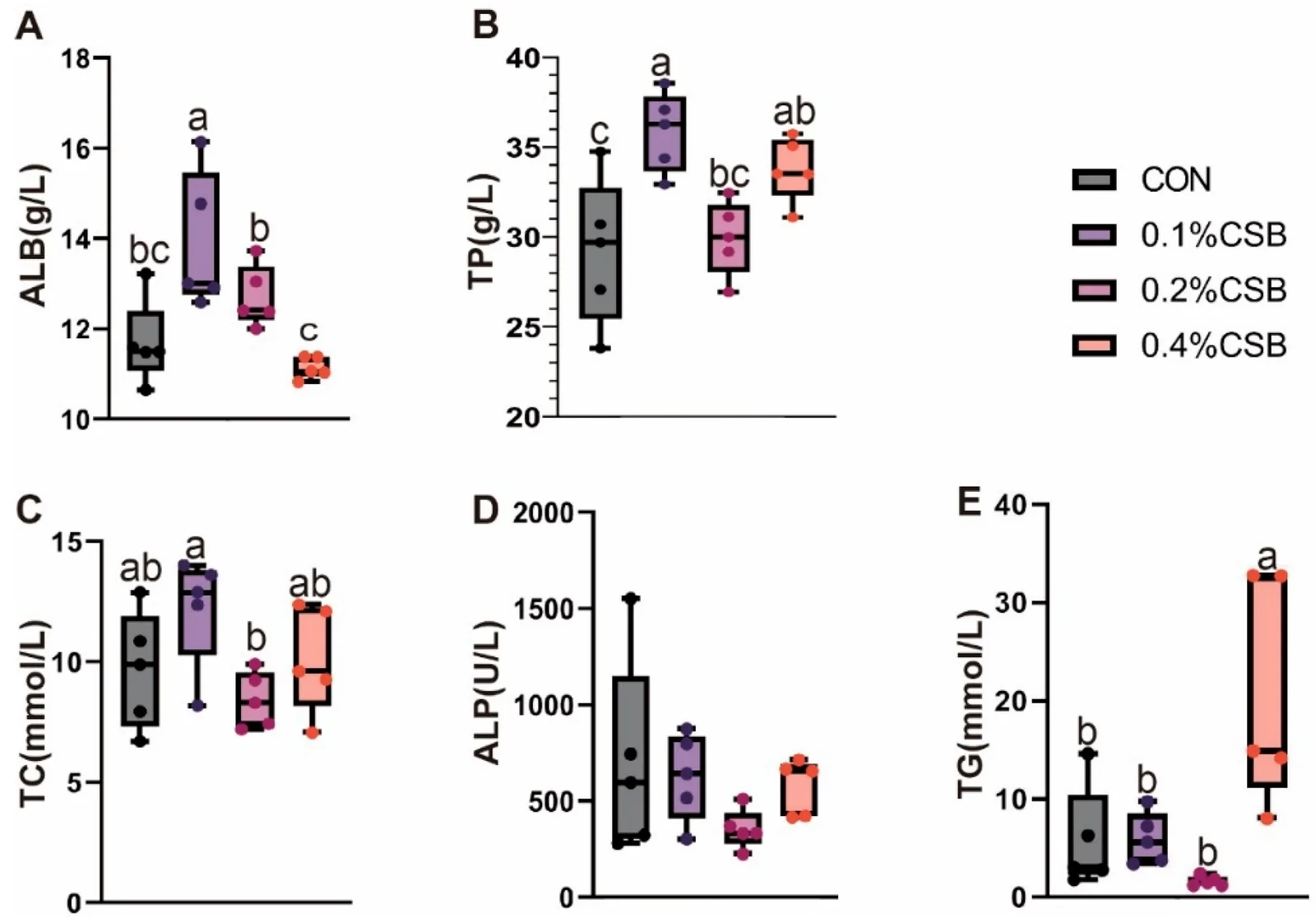
Effects of CSB on serum (**A**–**E**) biochemical analysis of pigeons. Data represent mean ± SE (*n* = 5), and bars with different letters (a, b, c) analyzed by one-way ANOVA followed by Duncan’s multiple range test among 4 groups differ significantly (*p* < 0.05); *p* values indicate significance. Control, pean-corn-sorghum and wheat; 0.1% CSB, 100 g/kg CSB; 0.2% CSB, 200 g/kg CSB; 0.4% CSB, 400 g/kg CSB. Abbreviations: ALB, serum albumin; TP, total protein; TC, total serum cholesterol; ALP, alkaline phosphatase; TG, triglyceride.

The serum ALP of pigeons at 5-months old did not differ significantly among four groups (*p* > 0.05; Figure 1D). Based on the results of the one-way ANOVA, it was found that pigeons in the 0.1% CSB group had the highest concentrations of ALB and TG in serum (*p* < 0.05; Figure 1A,E). In the main effect analysis, it was found that the 0.1% CSB group upregulated the level of TP in serum (*p* < 0.05; Figure 1B). The 0.2% CSB group had a lower level of ALB, TP and TC (*p* < 0.05; Figure 1A–C).

### 3.3. The Effects of CSB on the IOI

**Figure 2:**
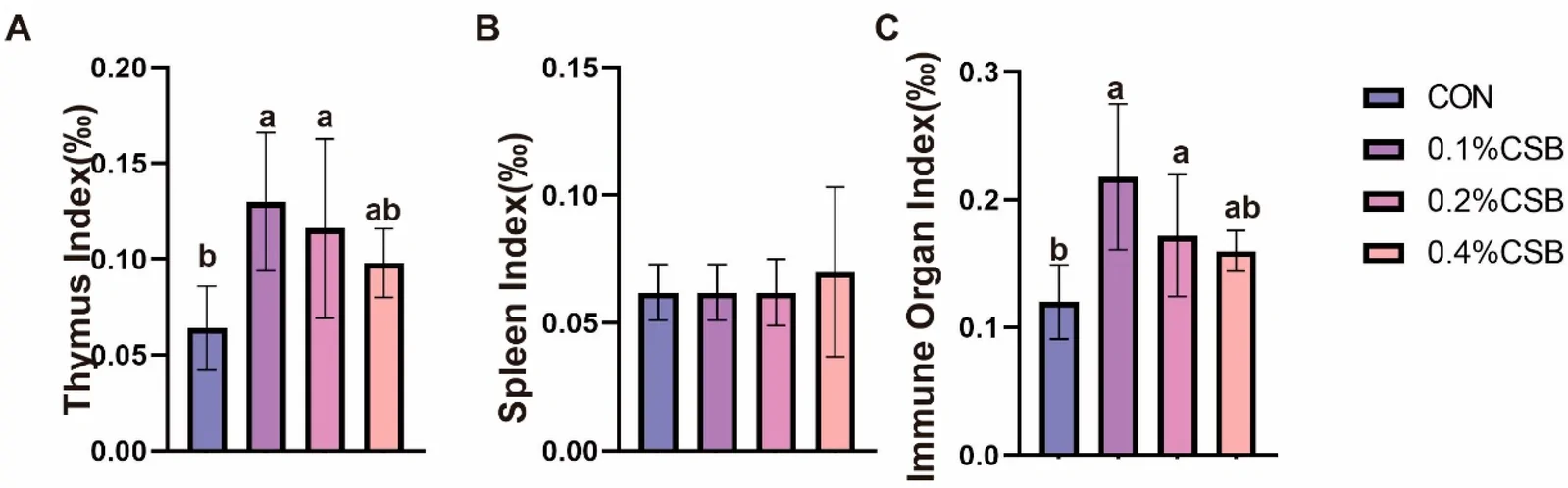
Effects of CSB on IOI (**A**–**C**) in pigeons. Data represent mean ± SE (*n* = 5), and bars with different letters (a, b) analyzed by one-way ANOVA followed by Duncan’s multiple range test among 4 groups differ significantly (*p* < 0.05). Abbreviations: Control, pean-corn-sorghum and wheat; 0.1% CSB, 100 g/kg CSB; 0.2% CSB, 200 g/kg CSB; 0.4% CSB, 400 g/kg CSB.

There is no significance in ALB of pigeons among the four groups (*p* > 0.05; Figure 2B), but the 0.1% CSB and 0.2% CSB groups showed a higher Thymus Index and IOI than the Control group (*p* < 0.05; Figure 2A,C).

### 3.4. Inflammatory Factors

**Figure 3:**
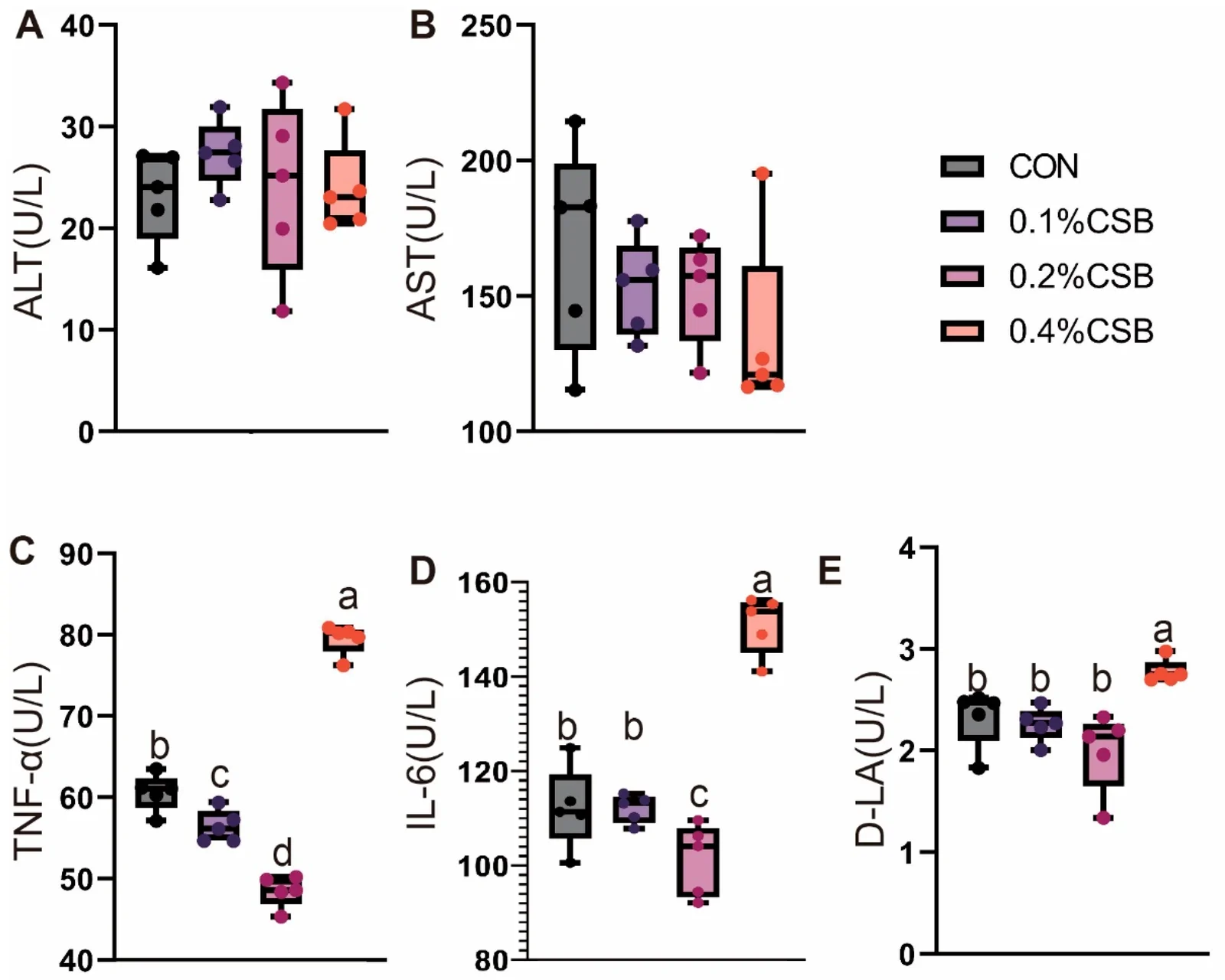
Effects of CSB on inflammatory factors (**A**–**E**) in pigeons. Data represent mean ± SE (*n* = 5), and bars with different letters (a, b, c, d) analyzed by one-way ANOVA followed by Duncan’s multiple range test among 4 groups differ significantly (*p* < 0.05), *p* values means the significant. Control, pean-corn-sorghum and wheat; 0.1% CSB, 100 g/kg CSB; 0.2% CSB, 200 g/kg CSB; 0.4% CSB, 400 g/kg CSB; Abbreviations: Interleukin-6 (IL-6), Tumor necrosis factor-α (TNF-α), D-Lactic acid (DL-A).

ALT and AST levels did not differ significantly between CSB and Control groups (*p* > 0.05; Figure 3A,B), according to the main effect analysis (Figure 3). The 0.4% CSB group had the highest serum concentrations of TNF-α, IL-6 and D-LA among the four groups (*p* < 0.05; Figure 3C–E). TNF-α and IL-6 concentrations in pigeons in the 0.2% CSB group were lower than in the other groups (*p* < 0.05; Figure 3C,D).

### 3.5. Intestinal Morphology

**Figure 4:**
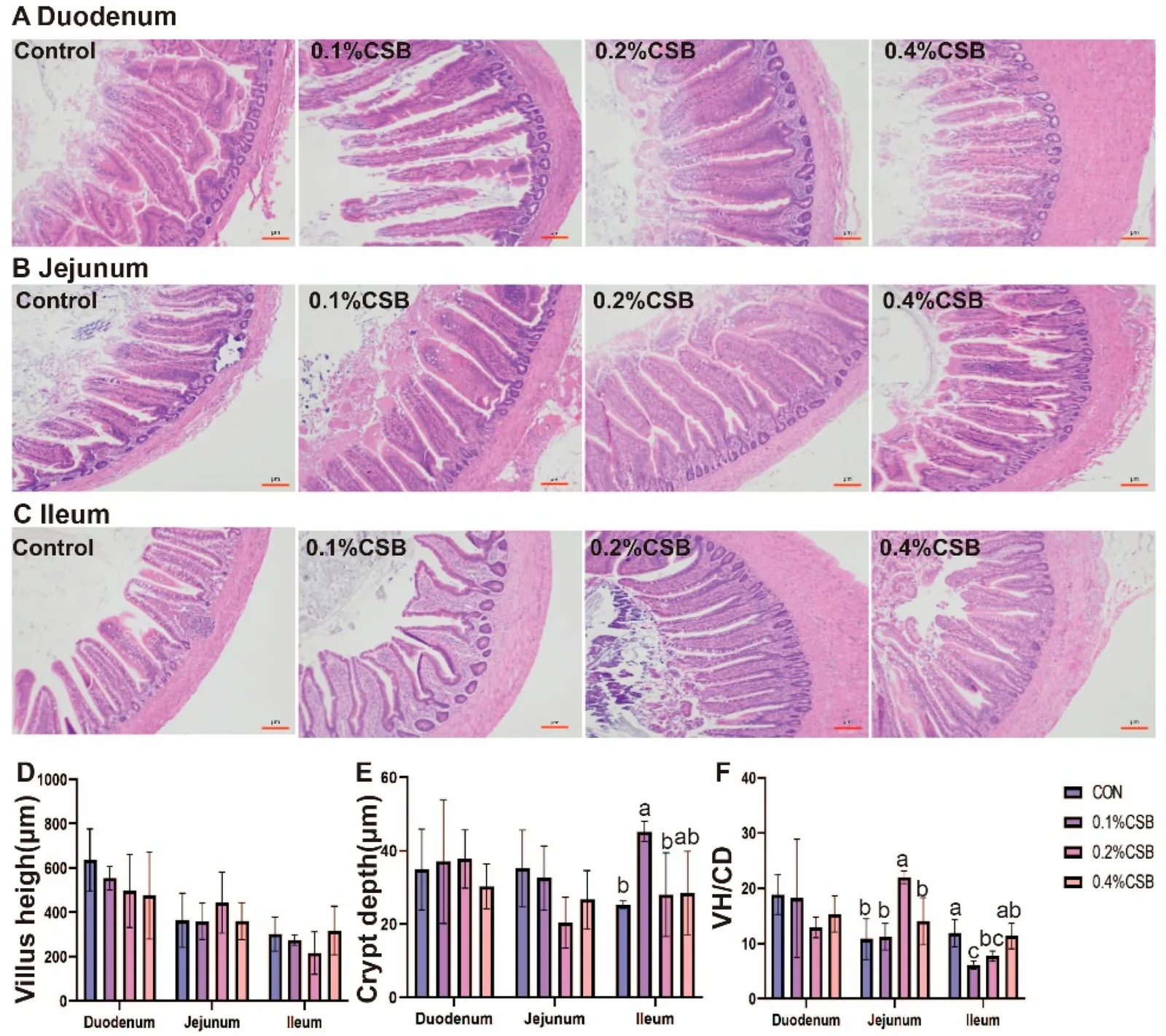
Effect of CSB on small intestinal histomorphology in young pigeons. The pictures of periodic acid-schiff-stained sections of duodenum (**A**, ×100), jejunum (**B**, ×100) and ileum (**C**, ×100), and villus height, crypt depth and villus height/crypt depth of duodenum (**D**–**F**). Data represent mean ± SE, and bars with different letters (a, b, c) analyzed by one-way ANOVA followed by Duncan’s multiple range test among 4 groups differ significantly (*p* < 0.05), *p* values means the significant. Control, pean-corn-sorghum and wheat; 0.1% CSB, 100 g/kg CSB; 0.2% CSB, 200 g/kg CSB; 0.4% CSB, 400 g/kg CSB.

5-month-old pigeons exhibit different morphological structures in the duodenum, jejunum, and ileum (Figure 4A–C). The three CSB groups showed no significant differences in duodenum, jejunum, and ileum VH (*p* > 0.05; Figure 4D), duodenum, jejunum CD (*p* > 0.05; Figure 4E), and duodenum VH/CD (*p* > 0.05; Figure 4F). However, the ileum CD in the 0.1% CSB group was significantly higher (*p* < 0.05; Figure 4E), and the VH/CD of the jejunum in the 0.2% CSB group was significantly higher (*p* < 0.05; Figure 4F).

### 3.6. Ileum Microbial Diversity

**Figure 5:**
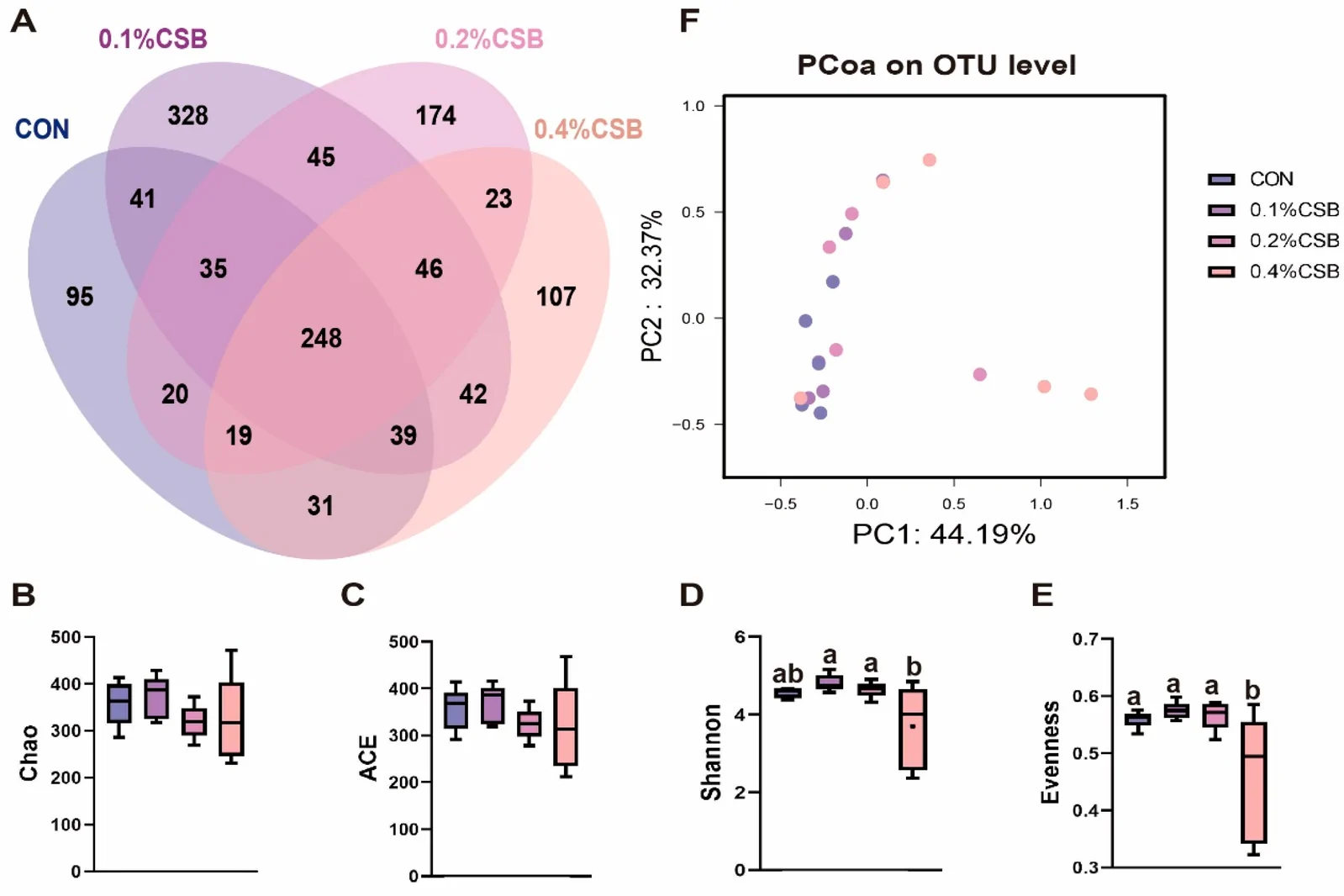
Effects of CSB on ileum microbial diversity (**A**–**F**) in young pigeons. The Venn diagram (**A**), box plots of α-diversity as measured by Chao index (**B**), ACE index (**C**), Shannon index (**D**) and Evenness index at OTU level (**E**), and PCoA plot (**F**) of the ileum microbiome based on unweighted-unifrac distance metrics at OTU level. Data (**B**–**E**) represent the mean and range from minimum to maximum, and bars with different letters (a, b) analyzed by one-way ANOVA followed by Duncan’s multiple range test among 4 groups differ significantly (*p* < 0.05); *p* values mean the significance. Control, pean-corn-sorghum, and wheat; 0.1% CSB, 100 g/kg CSB; 0.2% CSB, 200 g/kg CSB; 0.4% CSB, 400 g/kg CSB (*n* = 5, 0.2% CSB *n* = 4).

The ileum bacterial community was inspected using 16S rRNA gene sequences. Based on the Venn diagram (Figure 5A), 248 co-owned OTUs clustered into four groups (97% similarity level). The unique OTUs of the Control, 0.1% CSB, 0.2% CSB, and 0.4% CSB group pigeons were 95, 328, 174, and 107. The 0.4% CSB group had the lowest Shannon and evenness indices compared with the Control group (*p* < 0.05; Figure 5E). Furthermore, the ANOSIM test revealed microbiota community shifts at the OTU level across four groups (Figure 5F) in addition to PCoA based on unweighted-unifrac distance metrics.

### 3.7. Composition of the Ileum Microbial Community

**Figure 6:**
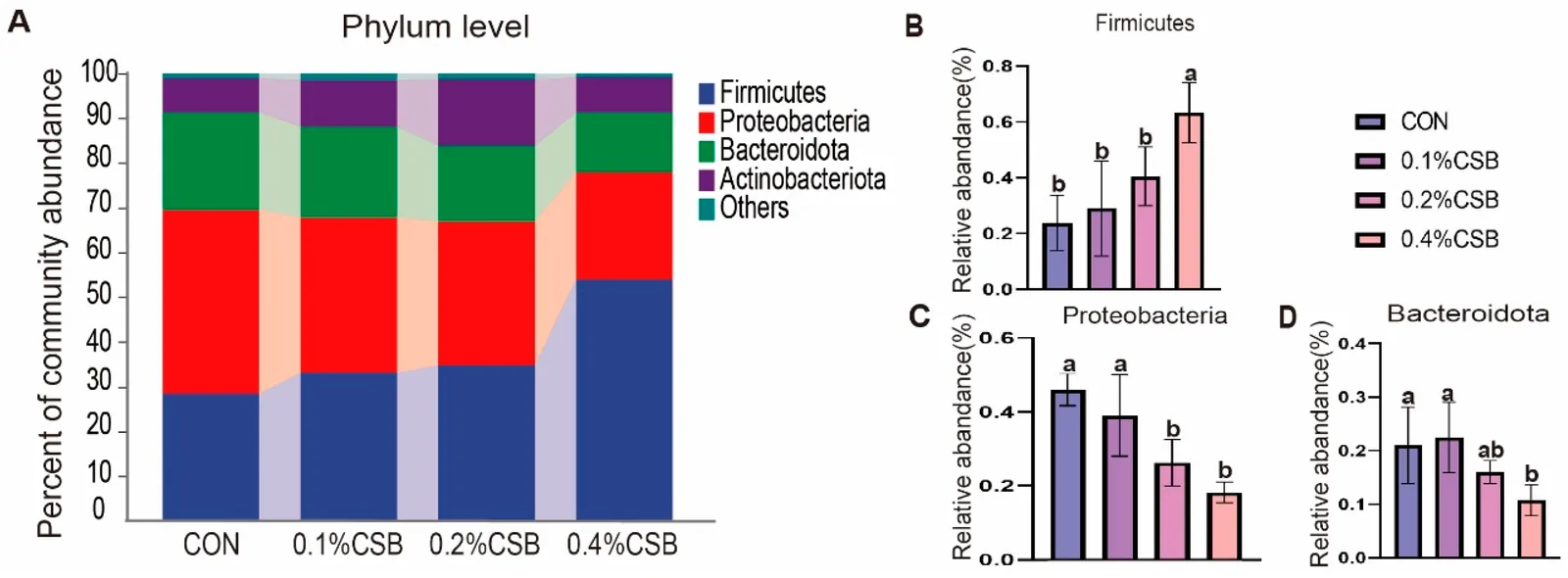
Effects of CSB on ileum microbial community compositions (**A**–**D**) in young pigeons. Community bar plot analysis at the phylum level (**A**). Comparative analysis of relative abundance of major bacteria at the phylum level (**B**–**D**). Data represent the mean and range from minimum to maximum, and bars with different letters (a, b) analyzed by one-way ANOVA followed by Duncan’s multiple range test among 4 groups differ significantly (*p* < 0.05); *p* values mean the significant. Control, pean-corn-sorghum and wheat; 0.1% CSB, 100 g/kg CSB; 0.2% CSB, 200 g/kg CSB; 0.4% CSB, 400 g/kg CSB (*n* = 5, 0.2% CSB *n* = 4).

An analysis of the microbial community at the phylum (Figure 6A) level was performed. Utilizing one-way ANOVA, Firmicutes were identified as the predominant phylum across all four groups, with a significantly higher relative abundance in the 0.4% CSB pigeon group compared to the other groups (*p* < 0.05; Figure 6B). Conversely, the relative abundance of Proteobacteria was significantly lower in the 0.2% CSB and 0.4% CSB groups relative to the remaining two groups (*p* < 0.05; Figure 6C). In addition, the main effect analysis demonstrated that CSB significantly decreased Bacteroidota relative abundance (*p* < 0.05; Figure 6D).

**Figure 7:**
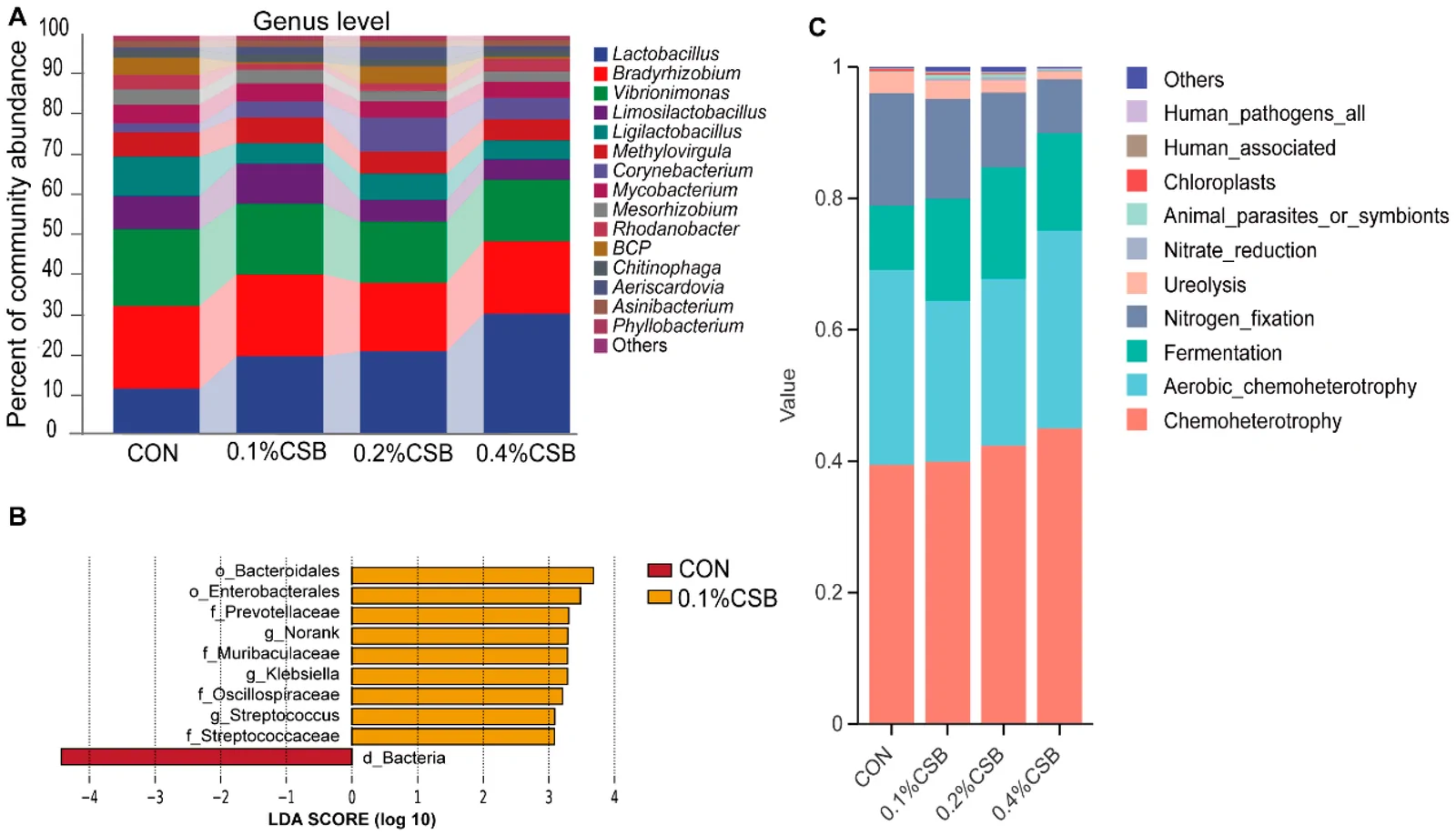
Effects of CSB on ileum microbial community compositions (**A**−**C**) in young pigeons. Community bar plot analysis at genus level (**A**). Histogram of the LDA value distribution (**B**). Functional Annotation of Prokaryotic Taxa (**C**). Control, pean-corn-sorghum and wheat; 0.1% CSB, 100 g/kg CSB; 0.2% CSB, 200 g/kg CSB; 0.4% CSB, 400 g/kg CSB (*n* = 5, 0.2% CSB *n* = 4).

At the genus level, the relative abundance of Lactobacillus successively increased with increasing concentrations of CSB in the Control group (10.44%), 0.1% CSB (16.84%), 0.2% CSB (18.01%), and 0.4% CSB (27.18%); the results were not statistically significant (*p* > 0.05; Figure 7A).

## 4. Discussion

Stress adversely affects production primarily by decreasing growth rates, reducing body weight (BW), and diminishing feed efficiency, while simultaneously increasing morbidity and mortality rates [11,12]. In pigeons, weaning stress poses a significant challenge that remains poorly understood. To evaluate whether CSB supplementation sustains growth performance and feed intake in pigeons, we examined the effects of dietary CSB on body weight and growth metrics in WK pigeons as indicators of physiological health. Specifically, 0.1% CSB supplementation significantly improved ADG and ADFI, possibly through immunological, intestinal, and microbiological mechanisms [13]. Previous studies have reported that CSB mitigates heat and oxidative stress in poultry and improves growth performance and intestinal microbiota in broilers [14,15,16,17]. However, excessive CSB supplementation may exert adverse effects in poultry [18]. Consistent with this observation, 0.4% CSB supplementation in the present study did not confer growth benefits. The growth-promoting effect of 0.1% CSB may be attributed to the multiple biological functions of butyrate. As a primary energy substrate for intestinal epithelial cells, butyrate supports epithelial renewal and mucosal integrity, thereby maintaining nutrient absorptive capacity [19].

Serum biochemical parameters serve as key indicators of health status, productivity, and physiological function in poultry [20,21]. TP and ALB exert humoral immunity and bacteriostatic effects, repair tissue damage, and enhance systemic immunity. Moreover, they reflect feed intake, metabolic status, and overall health [22,23]. ALP activity is a critical indicator of bone development and growth [6,24]. In the present research, TP and ALB contents were highest in the 0.1% CSB group, which is consistent with previous studies [20]. These results suggest that 0.1% CSB supplementation improves nutritional status and supports growth in pigeons. Butyrate may also regulate intestinal and systemic inflammatory responses through histone deacetylase inhibition and activation of G-protein-coupled receptors, including GPR41 and GPR43 [25]. These pathways can influence NF-κB-related signaling and the production of inflammatory cytokines such as TNF-α and IL-6 [26]. By moderating excessive inflammatory stimulation and supporting mucosal homeostasis, butyrate may indirectly contribute to immune organ development and improved physiological performance. This mechanism is consistent with the present observation that 0.2% CSB reduced TNF-α and IL-6 levels.

Growth performance in poultry is closely related to immune function. The spleen and thymus are key immune organs that confer disease resistance in poultry [13,27]. The thymus and spleen are sites of lymphocyte maturation and immune response activation; an increase in immune organ weight reflects enhanced immune function in poultry [28,29]. Studies have shown that CSB supplementation at 400 mg/kg enhances the thymic index in broilers [30,31], with additional evidence supporting its positive effect on the spleen index. Recent studies have demonstrated that CSB positively regulates intestinal barrier integrity and immune parameters in broilers [32]. In the present study, 0.1% and 0.2% CSB supplementation significantly improved the thymic index and immunological organ index, while TNF-α and IL-6 levels were reduced in the 0.2% CSB group. These findings are consistent with previous reports.

Growth performance in pigeons is closely linked to intestinal digestive and absorptive capacity [33]. In birds, nutrient digestion and absorption occur primarily in the small intestine, where villus height and the villus-to-crypt ratio determine absorptive surface area, influencing nutrient utilization and growth [34,35]. CSB supplementation has been shown to enhance villus height in turkeys and laying hens, as well as in pigs receiving butyrate supplements [36,37]. To examine these effects in pigeons, we evaluated small intestinal morphology across different levels of CSB supplementation. In 5-month-old WK pigeons, while no significant changes in VH, duodenal and jejunal CD, or duodenal VH/CD were observed across CSB groups, ileum CD in the 0.1% CSB group and jejunal VH/CD in the 0.2% CSB group were significantly elevated compared with the Control group. These results are partially consistent with previous findings in broilers showing that CSB enhances intestinal morphology [31], although the absence of significant VH changes in pigeons suggests that the morphological response to CSB may vary across avian species. The elevated jejunal VH/CD in the 0.2% CSB group indicates improved absorptive capacity, while the increased ileal CD in the 0.1% CSB group may reflect enhanced crypt cell proliferation supporting epithelial renewal. Notably, growth improvements were the combined effect of multiple factors, including intestinal morphology, barrier integrity, and microbiota modulation.

The intestinal tract serves as the primary site for feed digestion and nutrient absorption, and functions as the largest immune organ [38,39]. Among the factors regulating intestinal immunity, the microbiota could modulate immune responses and promote tolerance [40,41]. CSB modulates gut microbiota composition and refines intestinal morphology, promoting microbial balance. This action, in turn, mitigates epithelial injury, enhances intestinal development, and improves overall physiological function in poultry [15,42]. In this investigation, the diversity of ileal microbial communities was characterized by differences in the relative abundance of individual phyla and genera. Among the treatment groups, Firmicutes emerged as the predominant microbiota. Additionally, Bacteroidota, which plays a crucial role in the degradation of complex carbohydrates, exhibited a more sensitive response to CSB supplementation [43]. The enrichment of Oscillospiraceae and Muribaculaceae in 0.1% CSB-fed pigeons indicates that low-dose coated sodium butyrate favors fiber-fermenting, SCFA-producing taxa [37,44]. Muribaculaceae ferments dietary glycans into SCFAs, cross-feeds with Bifidobacterium and Lactobacillus, and is linked to protection against IBD, obesity, and diabetes [45]. This CSB-induced enrichment in pigeons extends prior broiler findings, suggesting the stimulatory effect of butyrate on fiber-fermenting taxa is evolutionarily conserved across avian hosts [37]. In the present study, the marked increase in nitrate reduction function, together with the concurrent shift in the abundance of facultative anaerobes such as Enterobacterales and Klebsiella, suggests that low-dose CSB may lower luminal oxygen tension in the pigeon gut, thereby shaping a microenvironment more conducive to the coexistence of facultative anaerobes and obligate anaerobic fermenters [46].

CSB supplementation exerts a beneficial effect on ileal microbiota composition, fosters the growth of advantageous bacterial populations, and facilitates microbiota regulation, thereby potentially alleviating the adverse effects associated with the weaning transition. Further research is warranted to elucidate the specific effects of the microbiota on WK pigeons in the context of the weaning transition.

## 5. Study Limitation

A limitation of the present study is that ileum microbiota composition was characterized solely by 16S rRNA sequencing, without functional analysis such as metagenomics or metabolomics. Although 16S rRNA profiling revealed shifts in microbial community structure, these data do not directly demonstrate the functional capacity of the microbiota or its causal role in weaning transition mitigation. In addition, the mechanisms by which CSB modulates immune function and intestinal barrier integrity were not assessed at the molecular level. Expression of intestinal barrier-related genes and key inflammatory pathway components was not measured, limiting our ability to elucidate the precise molecular mechanisms underlying the observed effects. Furthermore, the relatively small sample size (*n* = 4–5 per group) may reduce statistical power and limit the generalizability of the findings, particularly for subtle biological effects. Therefore, further studies incorporating metagenomic, metabolomic, or transcriptomic approaches with larger sample sizes are required to clarify the functional pathways through which CSB influences gut microbiota and host physiology in WK pigeons.

## 6. Conclusions

This study demonstrates that dietary CSB supplementation at 0.1% improves growth performance, serum biochemistry, immune organ development, and ileum microbiota composition in juvenile WK pigeons during the weaning transition stage. Specifically, 0.1% CSB increased ADG and ADFI, elevated serum TP and ALB levels, improved thymic index and the IOI, and enriched beneficial ileum bacteria, including Muribaculaceae and Oscillospiraceae.

Growth improvements were not attributable to digestive enzyme activity alone, as lipase, trypsin, and amylase activities decreased in CSB-treated groups, but rather resulted from combined effects on intestinal morphology, barrier integrity, and microbiota composition. These findings suggest that 0.1% CSB is the optimal supplementation level for WK pigeons, and that excessive CSB may disrupt metabolic homeostasis. Further studies are needed to clarify the mechanisms by which microbiota alterations mediate weaning transition mitigation in pigeons.

## Figures and Tables

**Table 1:** Composition and nutrient levels of the basal diet (air-dry basis)%.

Ingredients	Content	Nutrient Levels^2^	Content
Corn	53.00	ME/(MJ/kg)	12.81
Pea	30.00	CP	12.90
Wheat	7.00	Lys	1.05
Sorghum	7.00	Met	0.44
NaCl	0.50	Ca	1.20
Limestone	1.50	AP	0.54
Premix^1^	1.00		
Total	100.00		

^1^ The premix provided the following per kg of diets: VA 6000.00 IU, VD_3_ 1200.00 IU, VE 34.00 IU, VB_1_ 3.2 mg, VB_2_ 7.20 mg, VB_12_ 0.10 mg, biotin 1.34 mg, folic acid 0.55 mg, nicotinic acid 15.00 mg, pantothenic acid 7.50 mg, Cu (as copper sulfate) 12.00 mg, Fe (as ferrous sulfate) 35.00 mg, Zn (as zinc sulfate) 35.00 mg, Mn (as manganese sulfate) 55.00 mg, I (as potassium iodide) 0.25 mg. ^2^ Values are calculated.

**Table 2:** Effects of coated sodium butyrate on growth performance of pigeons.

Item	Control	0.1% CSB	0.2% CSB	0.4% CSB
BW				
Initial (g)	370.83 ± 5.21	343.75 ± 14.22	347.27 ± 16.30	371.13 ± 2.10
Final (g)	413.80 ± 7.19 ^b^	451.00 ± 16.70 ^a^	396.60 ± 18.90 ^b^	413.00 ± 4.14 ^b^
ADG (g/d)	0.36 ± 0.06 ^b^	0.89 ± 0.14 ^a^	0.41 ± 0.16 ^b^	0.35 ± 0.34 ^b^
ADFI				
Initial (g/d)	25.92 ± 2.65	25.21 ± 3.35	27.29 ± 3.56	24.85 ± 3.23
Final (g/d)	41.82 ± 1.26 ^b^	43.36 ± 1.34 ^a^	43.24 ± 1.77 ^a^	42.55 ± 1.56 ^b^

Abbreviations: a, b, superscripts for means belong to one-way ANOVA following by Duncan’s multiple range test among 4 groups (*n* = 5).

## Data Availability

The data presented in this study are available on request from the corresponding author, because the research is ongoing and the full dataset has not yet been finalized for public dissemination.

## Abbreviations

The following abbreviations are used in this manuscript:

DFI	Average daily feed intake
AGPs	Antimicrobial growth promoters
ALB	Albumin
CD	Crypt depth
CSB	Coated sodium butyrate
DL-A	D-lactic acid
ELISA	Enzyme-linked immunosorbent assay
IL-6	Interleukin-6
IOI	Immune organ index
TC	Total cholesterol
TG	Triglycerides
TNF-α	Tumor necrosis factor-α
TP	Total protein
VH	Villus height
VH/CD	Villus height to crypt depth ratio
WK pigeon	White King pigeon
